# Neutrophil‐to‐Lymphocyte Ratio as a Complementary Marker for Stroke‐Associated Pneumonia Risk in Acute Ischemic Stroke

**DOI:** 10.1155/srat/1182945

**Published:** 2026-09-10

**Authors:** Lisda Amalia, Nadiah Nurul Ikhsani

**Affiliations:** ^1^ Department of Neurology, Medical Faculty, Universitas Padjadjaran/RSUP Dr. Hasan Sadikin, Bandung, Indonesia

**Keywords:** acute ischemic stroke, dysphagia, neutrophil-to-lymphocyte ratio, NIHSS, stroke-associated pneumonia

## Abstract

**Background:**

One of the most common nonneurological complications after stroke is stroke‐associated pneumonia (SAP), which significantly increases morbidity and mortality. Early identification of factors associated with SAP is essential, with the neutrophil‐to‐lymphocyte ratio (NLR) emerging as a practical and accessible systemic inflammatory biomarker for clinical screening. This study is aimed at determining the incidence of SAP and analyzing the biological and mechanical factors influencing its occurrence in patients with acute ischemic stroke.

**Method:**

This retrospective cohort study included consecutive patients with acute ischemic stroke whose data were extracted from medical record reviews, including admission NLR. Bivariate analyses were performed to identify factors associated with SAP, followed by multivariable logistic regression to determine independent predictors. The diagnostic performance of NLR was evaluated using receiver operating characteristic (ROC) curve analysis.

**Results:**

The incidence of SAP among 615 acute ischemic stroke patients was 19.7%. Multivariable logistic regression identified NLR > 5.21, dysphagia, NIHSS > 8, age > 60 years, and nutritional risk as independently associated with SAP. NLR > 5.21 had the largest adjusted odds ratio (aOR 11.43, 95% CI 5.74–18.93). In ROC analysis, NLR demonstrated good discrimination (AUC 0.78), although NIHSS showed higher discrimination (AUC 0.84).

**Conclusions:**

NLR > 5.21 was independently associated with SAP and demonstrated good discriminatory performance. However, NIHSS showed superior discrimination, indicating that NLR should be considered as a complementary biomarker rather than a standalone predictor.

## 1. Introduction

Stroke remains a major global health burden, ranking as the second leading cause of death and third leading cause of disability worldwide. In Indonesia, it is the top cause of mortality, surpassing ischemic heart disease and diabetes mellitus. National surveys indicate a rising stroke prevalence, from 7 per 1000 in 2013 to 10 per 1000 in 2018, driven by increasing modifiable risk factors [1–3]. Ischemic stroke is the most common subtype, accounting for over 80% of global stroke cases and 67% in Indonesia [4, 5]. Advances in the understanding of ischemic stroke pathophysiology over the past decade have contributed to more precise classification and management, yet complications such as infection remain a significant challenge in acute stroke care. One of the most frequent and severe nonneurological complications of stroke is pneumonia, specifically stroke‐associated pneumonia (SAP), which typically develops within the first week following stroke onset [6–9].

SAP typically occurs within the first week after stroke onset and is linked to prolonged hospitalization, increased disability, and up to a threefold rise in mortality. Reported SAP prevalence ranges from 11% to 31% globally [8–10]. Local data from our institution indicate a rate of approximately 28%–29%, with significantly higher mortality in SAP cases (43.3% vs. 9.2%) [11, 12]. Early identification of high‐risk patients is crucial to improve outcomes, and inflammatory markers such as the neutrophil‐to‐lymphocyte ratio (NLR) have shown promise as accessible tools for SAP risk stratification [13, 14].

Although previous studies in our center have explored SAP across stroke types descriptively, the current study focuses specifically on acute ischemic stroke. We aim to identify key biological and mechanical independent predictors of SAP and evaluate the discriminative value of NLR as a predictive marker.

## 2. Methods

### 2.1. Study Design and Setting

This was a retrospective cohort study with consecutive sampling using secondary data obtained from medical records of patients with acute ischemic stroke admitted to the Neurology Inpatient Ward of Dr. Hasan Sadikin General Hospital, Bandung, Indonesia, between January 2021 and December 2024.

### 2.2. Participants

A total of 615 out of 1196 medical records were included based on inclusion and exclusion criteria. Eligible patients were aged ≥ 18 years, diagnosed with acute ischemic stroke by a neurologist confirmed through noncontrast head CT, and had complete clinical data including NLR on admission. Patients were excluded if they had underlying hematologic disorders, autoimmune disease, malignancy, COVID‐19, systemic infection other than respiratory, unclear pneumonia onset preceding stroke, and incomplete data. This study was approved by The Research Ethics Committee of Dr. Hasan Sadikin General Hospital Bandung.

### 2.3. Data Collection

Patient characteristics and clinical data were retrieved from medical records, including demographic variables (age, sex), comorbidities (hypertension, diabetes mellitus, heart disease, kidney disease, smoking history), nutritional risk according to the nutritional risk screening tool (MST) and patients were categorized as having nutritional risk according to the predefined MST threshold, National Institutes of Health Stroke Scale (NIHSS) score, history of prior stroke, lesion location (based on brain NCCT and TOAST classification), presence of dysphagia (clinical cranial nerve IX and X examination and water swallow test), and NLR at admission. The NLR was calculated from the first complete blood count obtained at hospital admission, before the diagnosis of SAP whenever this information was available. The interval between stroke onset and blood sampling was recorded when documented. Dysphagia was defined based on the documented bedside swallowing assessment performed during the acute hospitalization. The assessment included clinical evaluation of swallowing function and/or a water swallow test according to the institutional protocol. The timing of assessment and the professional responsible for the assessment were recorded when available. For patients with impaired consciousness who could not undergo a standard swallowing assessment, dysphagia status was determined according to the clinical documentation available in the medical record.

### 2.4. Outcome

The primary outcome was the occurrence of SAP within 7 days of stroke onset, diagnosed based on the Pneumonia in Stroke Consensus Group (PISCES) criteria. SAP was defined as the presence of [15]:

1. Pneumonia within first 7 days of stroke onset,
2. At least one of the following
  a. Fever > 38°C without other known etiology.
  b. Leukopenia < 4000/mm^3^ or leukocytosis > 12,000/mm^3^.
  c. Decreased level of consciousness without other cause (in patients > 70 years old).
3. At least two of the following:
  a. New onset purulent sputum within the past 24 h.
  b. New onset of cough or respiratory rate > 25x/min.
  c. Rales, crackles, or bronchial breath sounds; or
  d. Desaturation (PaO2/FiO2 ≤ 240, or increase oxygen demand).
4. Radiological evidence on chest x‐ray, based on ≥ 2 views showing at least one of the following:
  a. New or progressive infiltrate.
  b. Consolidation.
  c. Cavitation.

SAP was classified into [15]:

• Probable SAP: Clinical criteria were met without new or progressive findings on chest x‐ray and no alternative diagnosis to explain the condition.
• Definite SAP: Clinical criteria were met with radiological evidence of pneumonia on at least one chest x‐ray.

### 2.5. NLR Assessment

NLR was calculated from the absolute neutrophil and lymphocyte counts obtained from the admission complete blood count. Blood sampling was performed at the time of initial hospital assessment. Information regarding antibiotic or corticosteroid administration before blood sampling was not systematically captured in the study dataset; therefore, the potential influence of presampling medication exposure on leukocyte differential counts and NLR could not be reliably quantified or adjusted for in the analysis.

### 2.6. Statistical Analysis

Data were analyzed using the Statistical Package for Social Science (SPSS) Version 26.0 for Windows. Continuous variables were presented as mean ± standard deviation (SD) or median with interquartile range (IQR) based on distribution, and categorical variables were presented as frequencies and percentages. Bivariate analysis was conducted using chi‐square test or Fisher′s exact test for categorical variables and Mann–Whitney *U* test for continuous variables. Variables with *p* < 0.25 were included in multivariate analysis using binary logistic regression to identify independent predictors of SAP. The predictive performance of NLR was evaluated using receiver operating characteristic (ROC) curve analysis. A *p* value < 0.05 was considered statistically significant. For categorical variables, crude proportion ratios (PRs) with corresponding 95% confidence intervals (CIs) were estimated using modified Poisson regression with robust standard errors. A PR greater than 1 indicated a higher proportion of SAP among patients with the specified exposure, whereas a PR below 1 indicated a lower proportion. Statistical significance was defined as a two‐sided *p* value < 0.05.

### 2.7. Internal Validation

Internal validation was performed using nonparametric bootstrap resampling with 1000 iterations. To account for potential overfitting, an optimism‐corrected estimate of model performance (e.g., area under the curve [AUC]/c‐statistic) was calculated by subtracting the average optimism—defined as the mean difference between the performance in the bootstrap sample and that in the original dataset—from the apparent performance. The reported 95% CIs reflect the empirical percentile distribution (2.5th to 97.5th percentiles) across the bootstrap samples, representing the sampling variability of the estimated performance metrics.

### 2.8. Ethics Approval and Consent to Participate

This study was approved by the Ethics Committee of Dr. Hasan Sadikin General Hospital, Bandung, Indonesia (Ethical Clearance Number: DP.04.03/D.XIV.6.5/315/2024). Written informed consent was waived due to the retrospective nature of the study. This study complied with all relevant ethical regulations (including The Declaration of Helsinki).

## 3. Results

**Figure 1:**
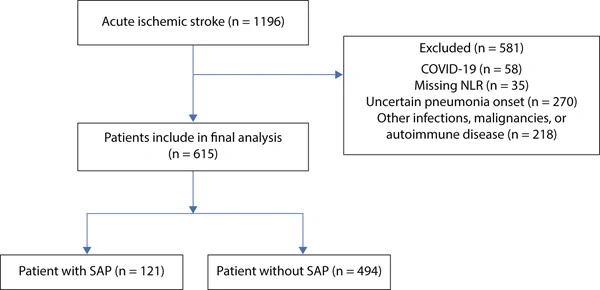
Flow diagram of patients recruitment.

Of the 1196 medical records initially screened, 615 patients met the predefined eligibility criteria and were included in the final analysis. The reasons for exclusion included incomplete laboratory data, unavailable NLR measurements, predefined clinical exclusion criteria, and/or incomplete documentation, as applicable (Figure 1).

### 3.1. Baseline Characteristics and Clinical Factors Associated With SAP

Among the 615 patients with acute ischemic stroke included in the analysis, 121 (19.7%) developed SAP, whereas 494 (80.3%) did not. Patients with SAP had a higher median age, higher admission NIHSS score, higher NLR, and longer hospital stay than those without SAP. The median age was 64 years (IQR, not shown) in the SAP group compared with 58 years in the non‐SAP group. The median admission NIHSS score was 13 in patients with SAP compared with 6 in those without SAP. Similarly, median NLR was higher among patients with SAP than among those without SAP (5.70 vs. 2.95). All three variables showed statistically significant between‐group differences (all *p* < 0.001).

Several clinical characteristics were also more frequently observed among patients who developed SAP. Dysphagia was present in 90 of 121 patients with SAP (74.4%) compared with 96 of 494 patients without SAP (19.4%), corresponding to a crude PR of 6.70. Kidney disease was present in 26 patients with SAP (21.5%) and 35 patients without SAP (7.1%), with a corresponding PR of 2.49. Nutritional risk was identified in 69 patients with SAP (57.0%) compared with 145 patients without SAP (29.4%), corresponding to a PR of 2.49. Smoking history was less frequent among patients with SAP than among those without SAP (38.0% vs. 51.2%; PR, 0.65).

For diabetes mellitus, 38 patients with SAP (31.4%) and 119 patients without SAP (24.1%) had diabetes, whereas the absence of diabetes was observed in 83 (68.6%) and 375 (75.9%) patients, respectively. The latter value was corrected from the previously reported 57.9% following verification of the original dataset.

Because the ratios presented in the descriptive comparison represent the ratio of the proportion of each characteristic between the SAP and non‐SAP groups, these estimates are reported as crude PRs rather than regression‐derived relative risks. Multivariable associations are reported separately using adjusted odds ratios from binary logistic regression (Table 1).

**Table 1:** Subject characteristics.

Variable	*N*(%)
Sex	
Male	352 (57.2)
Female	263 (42.8)
SAP	121 (19.7)
Probable	35 (28.9)
Definite	86 (71.1)
Biological factors	
Age	
Mean ± SD	58.9 ± 11.5
Comorbid factors	
Hypertension	578 (94.0)
Diabetes mellitus	157 (25.5)
Heart diseases	433 (70.4)
Kidney diseases	61 (9.9)
Smoking history	299 (48.6)
Nutritional risk	214 (34.8)
NLR	
Median (IQR)	3.14 (2.46)
Range	0.06–31.61
Mechanical factors	
History of stroke	
First stroke	312 (50.7)
Recurrent stroke	303 (49.3)
Stroke lesion location	
Cortical subcortical	181 (29.4)
Subcortical	365 (59.8)
*Midbrain*	1 (0.2)
Pons	64 (10.4)
Cerebellum	1 (0.2)
TOAST classification	
LAA	159 (25.9)
CE	83 (13.5)
SVO	373 (60.7)
NIHSS	
Median (IQR)	7.0 (7.0)
Range	2.0–23.0
Presence of dysphagia	186 (30.2)

*Note:* Percentages for SAP subtypes were calculated using the SAP subgroup (*n* = 121) as the denominator.

### 3.2. Bivariate Analysis

Bivariate analysis demonstrated that several clinical and laboratory characteristics were associated with SAP. Dysphagia showed the strongest association with SAP, with a crude PR of 6.70 (95% CI: 4.62–9.70; *p* < 0.001). Patients with nutritional risk had approximately 2.5‐fold higher proportions of SAP compared with those without nutritional risk (PR 2.49, 95% CI: 1.81–3.42; *p* < 0.001). Kidney disease was also associated with a higher proportion of SAP (PR 2.49, 95% CI: 1.76–3.51; *p* < 0.001).

Patients with NLR > 5.21 had a substantially higher proportion of SAP than those with NLR ≤ 5.21 (PR 5.76, 95% CI: 4.29–7.74; *p* < 0.001). In contrast, smoking history was associated with a lower proportion of SAP (PR 0.65, 95% CI: 0.47–0.90; *p* = 0.010). Recurrent stroke, Type 2 diabetes, and cardiac disorders showed borderline associations with SAP, whereas sex and hypertension were not significantly associated with SAP. Nutritional risk was identified in 34.8% of patients according to the MST.

Both lesion location and TOAST classification were significantly associated with SAP (*p* < 0.0001). Cortical‐subcortical lesions were more common in the SAP group, whereas subcortical lesions predominated in the non‐SAP group. In terms of stroke etiology, SAP was more frequent in patients with cardioembolic stroke (51.8%) and large artery atherosclerosis (46.5%), and was rare in small vessel occlusion (1.1%).

Stroke severity, as measured by the NIHSS, was significantly higher in the SAP group (median 13.0 [IQR 7.0]) than in the non‐SAP group (median 6.0 [IQR 5.0]) (*p* < 0.0001) (Table 2).

**Table 2:** Bivariate analysis.

Variables	SAP (*N* = 615)		Ratio of proportion/PR	95% CI	*p*
	None (*n* = 494)	Present (*n* = 121)			
Sex					
Male	282 (57.1)	70 (57.9)	0.98	0.71–1.35	0.879^a^
Female	212 (42.9)	51 (42.1)			
Biological factors					
Age (years)					
Mean ± SD	57.5 ± 11.2	64.5 ± 11.3			< 0.00001^a^ ^∗^
Hypertension, *n*/*N* (%)	465/494 (94.1)	113/121 (93.4)	0.90	0.48–1.71	0.756^a^
Diabetes mellitus, *n*/*N* (%)	119/494 (24.1)	38/121 (31.4)	1.30	0.95–1.87	0.094^a^ ^∗∗^
Heart disease, *n*/*N* (%)	339/494 (68.6)	94/121 (77.7)	1.46	0.99–2.16	0.057^a^ ^∗∗^
Kidney disease, *n*/*N* (%)	35/494 (7.1)	26/121 (21.5)	2.49	1.76–3.51	< 0.0001^a^ ^∗^
Smoking history, *n*/*N* (%)	253/494 (51.2)	46/121 (38.0)	0.65	0.47–0.90	0.010^a^ ^∗∗^
Nutritional risk *n*/*N* (%),	145/494 (29.4)	69/121 (57.0)	2.49	1.81–3.42	< 0.0001^a^ ^∗^
NLR					
Median (IQR)	2.95 (1.96)	5.7 (6.3)	5.76	4.29–7.74	< 0.0001^a^ ^∗^
Mechanical factors					
History of stroke, *n*/*N* (%)	234/494 (47.4)	69/121 (57.0)	1.37	0.99–1.89	0.059
Lesion location					< 0.0001^a^ ^∗^
Cortical subcortical	108 (29.1)	73 (60.3)			
*Midbrain*	1 (0.2)	0			
Pons	50 (10.1)	14 (11.6)			
Cerebellum	1 (0.2)	0			
Subcortical	334 (67.6)	34 (28.1)			
TOAST classification					
LAA	85 (53.5)	74 (46.5)			< 0.0001^a^ ^∗^
CE	40 (48.2)	43 (51.8)			
SVO	369 (98.9)	4 (1.1)			
NIHSS					
Median (IQR)	6.0 (5.0)	13.0 (7.0)			< 0.0001^a^ ^∗^
Presence of dysphagia, *n*/*N* (%)	96/494 (19.4)	90/121 (74.4)	6.70	4.62–9.70	< 0.0001^a^ ^∗^

Abbreviations: CI, confidence interval; NLR, neutrophil‐to‐lymphocyte ratio; PR: proportion ratio.

### 3.3. ROC Analysis

**Figure 2:**
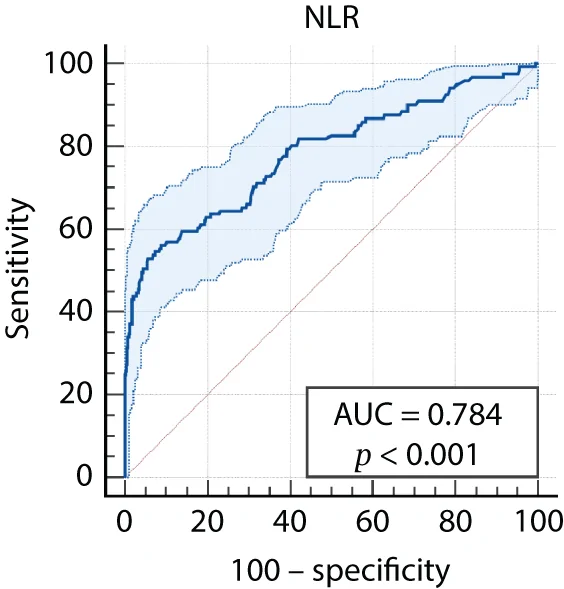
ROC curve of NLR.

ROC analysis showed that the NLR had an AUC of 0.78 (95% CI: 0.74–0.81) (Figure 2). At an NLR threshold of > 5.21, the sensitivity was 56.2% and specificity was 91.1%. Although the discriminatory performance of NLR was moderate, its AUC was lower than that of the NIHSS, which demonstrated an AUC of 0.84. Therefore, NLR should not be described as having the highest discriminatory ability among the evaluated parameters. NIHSS demonstrated better discrimination than NLR, although NLR showed good discriminatory performance. A summary of cutoff points, AUC, sensitivity, and specificity is provided in Table 3.

**Table 3:** Cutoff values, AUC, sensitivity, and specificity for age, NIHSS, and NLR based on ROC analysis.

Variables	Cutoff	AUC (CI 95%)	Sensitivity (CI 95%)	Specificity (CI 95%)
Age (years)	> 60	0.66 (0.62–0.70)	66.1 (57.0–74.5)	60.5 (56.1–64.9)
NIHSS	> 8	0.84 (0.80–0.86)	80.9 (72.9–87.6)	71.6 (67.5–75.6)
NLR	> 5.21	0.78 (0.74–0.81)	56.2 (46.9–65.2)	91.1 (88.2–93.5)

### 3.4. Diagnostic Performance of NLR

The diagnostic performance of NLR for SAP was further evaluated using a threshold of > 5.21. (Table 4). At this cutoff, 68 of 121 patients with SAP had an NLR above the threshold, whereas 53 had an NLR at or below the threshold. Among patients without SAP, 44 had an NLR > 5.21 and 450 had an NLR ≤ 5.21. The resulting sensitivity was 56.2% (95% CI, 46.9%–65.2%), whereas specificity was 91.1% (95% CI, 88.2%–93.5%). The positive predictive value was 60.7% (95% CI, 51.0%–69.8%), and the negative predictive value was 89.5% (95% CI, 86.4%–92.0%). Overall, diagnostic accuracy was 84.2% (95% CI, 81.1%–87.0%).

**Table 4:** Diagnostic performance of NLR.

Parameter	Estimate	95% CI
Sensitivity	56.2%	46.9%–65.2%
Specificity	91.1%	88.2%–93.5%
PPV	60.7%	51.0%–69.8%
NPV	89.5%	86.4%–92.0%
Accuracy	84.2%	81.1%–87.0%

These findings indicate that an NLR threshold of > 5.21 had relatively high specificity and negative predictive value but only moderate sensitivity for identifying SAP. Therefore, NLR may be more useful as a supportive marker for identifying patients at lower probability of SAP rather than as a stand‐alone screening test for all cases.

### 3.5. Multivariate Analysis

Multivariate logistic regression was performed using variables with *p* < 0.25 in bivariate analysis and clinical relevance. Five independent predictors of SAP were identified and the complete results of the multivariate regression model are provided in Table 5. Patients with NLR > 5.21 had approximately 11‐fold higher odds and strongest association of SAP.

**Table 5:** Multivariable logistic regression analysis of factors associated with stroke‐associated pneumonia.

Variables	Coefficient (B)	Standard error	Statistic Wald	Adjusted odds ratio (aOR)	CI 95%	*p*
Age (> 60 years old)	1.319	0.289	20.812	3.87	2.12–6.59	**< 0.0001** ^∗^
NIHSS (> 8)	1.454	0.321	20.459	4.39	2.28–8.03	**< 0.0001** ^∗^
Dysphagia	1.534	0.324	22.424	4.46	2.54–8.48	**< 0.0001** ^∗^
Nutritional risk	1.015	0.278	13.323	2.80	1.60–4.75	**< 0.0001** ^∗^
NLR (> 5.21)	2.342	0.304	59.409	11.43	5.74–18.93	**< 0.0001** ^∗^

Abbreviations: aOR, adjusted odds ratio; CI, confidence interval; NLR, neutrophil‐to‐lymphocyte ratio.

### 3.6. Internal Validation

Among 615 patients, 121 (19.7%) developed SAP. The apparent five‐predictor model showed an AUC of 0.918 and a Brier score of 0.086. After 1000 bootstrap repetitions, mean optimism was 0.005 for the AUC, 0.003 for the Brier score, 0.021 for the calibration intercept, and 0.035 for the calibration slope (Supporting Information). The optimism‐corrected AUC was 0.913, whereas the optimism‐corrected Brier score was 0.090. The optimism‐corrected calibration intercept was −0.021, and the calibration slope was 0.965. The resulting uniform shrinkage factor of 0.965 indicates relatively limited overfitting within the derivation cohort (Table 6).

**Table 6:** Bootstrap internal validation of the multivariable SAP model.

Performance measure	Apparent	Mean optimism	Optimism‐corrected	Bootstrap test‐performance 95% CI
AUC (C‐statistic)	0.918	0.005	0.913	0.911–0.919
Brier score	0.086	0.003	0.090	0.086–0.091
Calibration intercept	0.000	0.021	−0.021	−0.322–0.296
Calibration slope	1.000	0.035	0.965	0.800–1.144

*Note:* Lower Brier scores indicate better overall predictive accuracy. Calibration intercept values closer to 0 and calibration slopes closer to 1 indicate better calibration. The confidence intervals are the 2.5th and 97.5th percentiles of the bootstrap test‐performance distribution.

## 4. Discussion

This study identified key clinical predictors of SAP in patients with acute ischemic stroke. SAP occurred in 19.7% of cases, with the majority categorized as definite SAP. The findings reinforce the multifactorial nature of SAP, involving both biological and mechanical risk factors. The principal finding of this study was that elevated NLR was independently associated with SAP after adjustment for age, neurological severity, dysphagia, and nutritional risk.

### 4.1. Biological Factors

Among the biological variables evaluated, older age (> 60 years), nutritional risk, and elevated NLR were significantly associated with SAP. Older age remained an independent predictor in multivariate analysis (aOR 3.87; 95% CI: 2.12–6.59), aligning with prior studies that highlight age‐related immune decline and swallowing dysfunction as contributors to poststroke infections. [11, 12, 16–21]. Nutritional risk, identified through routine nutritional screening, also showed a strong association with SAP (aOR 2.80; 95% CI: 1.60–4.75), reflecting its role in impairing host defenses. Patients with nutritional risk tend to experience delayed rehabilitation, reduced dietary intake, and more severe dysphagia. These conditions weaken immune response and decrease respiratory defense mechanisms, increasing the risk of pulmonary infection [22–24].

NLR was the only inflammatory biomarker assessed in this study and emerged as the most dominant predictor of SAP and the factor with the largest adjusted odds ratio. An NLR > 5.21 was associated with an 11‐fold increased risk of SAP (aOR 11.43; 95% CI: 5.74–18.93). This finding supports previous evidence that elevated NLR reflects an imbalance between proinflammatory and adaptive immune responses, contributing to increased infection susceptibility [13, 14, 16, 25]. With its accessibility and low cost, NLR may serve as a practical screening tool for early SAP risk stratification. Because the retrospective dataset did not allow precise standardization of the interval between stroke onset and blood sampling in all patients, temporal variation in NLR measurement may have introduced measurement heterogeneity.

Other comorbidities such as kidney disease, diabetes mellitus, and heart disease showed associations in bivariate analysis, but were not retained in the final model—potentially due to interaction with other variables such as age and stroke severity. These conditions may contribute to SAP risk through mechanisms such as immune dysregulation in diabetes, uremic toxin accumulation in chronic kidney disease [26–28], diabetes through hyperglycemia‐induced neutrophil dysfunction [16, 29, 30], and chronic sympathetic overdrive inducing sustained catecholamine production which may dysregulate immune responses and increase susceptibility to infection poststroke in cardiac dysfunction [31–33].

### 4.2. Mechanical Factors

Stroke severity was strongly associated with SAP, with NIHSS > 8 increasing risk over fourfold (aOR 4.39, 95% CI: 2.28–8.03). Higher NIHSS scores reflect impaired consciousness, dysphagia, and reduced mobility—all contributing to aspiration risk [13, 34]. Dysphagia itself was the mechanical predictor (aOR 4.46, 95% CI: 2.54–8.48), reinforcing the need for early swallowing assessments. Patients with impaired swallowing function have a markedly increased risk of aspirating food, liquids, or secretions into the respiratory tract, predisposing them to lower respiratory tract infections, including SAP [13, 34–37].

The ROC analysis demonstrated that NIHSS had greater discriminatory ability than NLR. This finding is clinically plausible because NIHSS captures neurological impairment, which is closely related to dysphagia, impaired consciousness, and aspiration risk, whereas NLR reflects systemic inflammatory and immune responses. Therefore, NLR may provide complementary biological information rather than replace established clinical severity assessment. NLR reflects biological/inflammatory vulnerability, whereas NIHSS captures neurological severity and indirectly aspiration risk [37].

Stroke may promote SAP through the interaction of systemic inflammation and impaired airway protection. Acute ischemic stroke can induce a systemic inflammatory response characterized by neutrophilia and lymphopenia, resulting in an increased NLR and impaired immune competence. Concurrently, neurological dysfunction, reflected by higher NIHSS scores and dysphagia, compromises airway protection and increases the risk of aspiration. The convergence of impaired host defense and aspiration‐related pulmonary exposure may therefore increase susceptibility to SAP [34–37].

### 4.3. Internal Validation

The bootstrap analysis provides evidence that the SAP prediction model retained good performance after correction for optimism. The apparent AUC of 0.918 decreased only modestly to an optimism‐corrected AUC of 0.913, with mean optimism of 0.005. This relatively small optimism suggests that the discrimination observed in the derivation cohort was not substantially driven by overfitting. Internal validation is particularly important in a retrospective single‐center dataset because the same observations are used for model estimation and initial assessment of performance.

Calibration was also preserved after bootstrap correction. The calibration intercept was −0.021, close to the ideal value of zero, whereas the calibration slope was 0.965, close to the ideal value of one. The corresponding shrinkage factor of 0.965 indicates only modest regression‐to‐the‐mean correction of the model coefficients. Taken together, these findings suggest that the model combines adequate discrimination with acceptable calibration within the development population.

The model is clinically coherent because it combines different dimensions of SAP susceptibility: neurological severity (NIHSS), impaired airway protection (dysphagia), biological/inflammatory vulnerability (NLR), age‐related risk, and nutritional risk. This integrated approach is consistent with the manuscript′s proposed framework in which acute stroke‐related systemic inflammation and immune dysregulation coexist with impaired airway protection and aspiration susceptibility. NLR therefore contributes complementary biological information rather than replacing clinical assessment of stroke severity or swallowing function.

However, the bootstrap results should not be interpreted as evidence of external validity. Internal validation evaluates model stability within the development cohort and cannot determine whether the same coefficients, risk thresholds, calibration, or discrimination will be maintained in other hospitals or populations. External validation using an independent cohort is therefore required before clinical implementation. Prospective validation should also use standardized timing of NLR measurement and standardized dysphagia assessment. Internal bootstrap validation demonstrated that the multivariable model incorporating age > 60 years, NIHSS > 8, dysphagia, nutritional risk, and NLR > 5.21 retained good discriminative performance after correction for optimism (optimism‐corrected AUC 0.913). Calibration was acceptable, with an optimism‐corrected calibration intercept of −0.021 and calibration slope of 0.965. These findings support the internal robustness of the model but do not replace external validation. NLR should therefore be interpreted as a complementary inflammatory biomarker within a multidimensional SAP risk model rather than as a standalone predictor.

### 4.4. Implications

These findings highlight the importance of combining clinical severity, nutritional risk, and inflammatory markers, and swallowing assessment in early SAP risk stratification. NLR, in particular, offers practical utility as a predictive tool. NLR may be useful as an inexpensive and readily available adjunctive marker for early SAP risk stratification, particularly when interpreted alongside neurological severity and swallowing assessment. Elevated NLR is independently associated with SAP and provides complementary inflammatory information beyond established clinical risk factors, although neurological severity assessed by NIHSS demonstrates superior discriminatory performance. Prospective validation and implementation of structured screening protocols may improve outcomes in acute stroke care.

### 4.5. Strengths and Limitations

This study has several strengths. First, it included a relatively large cohort of 615 patients with acute ischemic stroke and used consecutive patient recruitment, thereby reducing the potential for selective inclusion. Second, NLR was assessed using routinely available admission laboratory parameters, supporting the clinical feasibility of this biomarker. Third, the analysis incorporated both clinical risk factors and NLR and included internal validation of the predictive model using bootstrap resampling.

Several limitations should also be considered. The retrospective, single‐center design limits the generalizability of the findings and introduces the possibility of selection and information bias. Although multiple clinically relevant covariates were incorporated into the analysis, residual confounding cannot be excluded. In particular, antibiotic and corticosteroid exposure before blood sampling was not systematically recorded and therefore could not be adequately adjusted for, despite the potential effects of these medications on circulating neutrophil and lymphocyte counts. In addition, the diagnostic performance of NLR was evaluated within the same study population, and external validation in an independent cohort is required before the proposed threshold can be applied broadly in clinical practice. Finally, prospective multicenter studies with standardized timing of blood sampling and systematic documentation of potential confounders are needed to confirm the reproducibility and clinical utility of the findings.

## 5. Conclusion

In this retrospective study, five independent predictors of SAP were identified: age > 60 years, NIHSS score > 8, dysphagia, nutritional risk, and NLR > 5.21. NLR > 5.21 is independently associated with SAP and has good discriminatory performance, but its discrimination is inferior to NIHSS; therefore, NLR should be regarded as a complementary inflammatory biomarker rather than a standalone predictive marker. NLR > 5.21 demonstrated the strongest independent association with SAP in the multivariable logistic regression model highlighting its potential as adjunctive risk stratification biomarker for early SAP risk stratification. Elevated NLR is independently associated with SAP and provides complementary inflammatory information beyond established clinical risk factors, although neurological severity assessed by NIHSS demonstrates superior discriminatory performance. Future prospective studies with standardized dysphagia evaluations and multicenter data are warranted to validate these results and improve SAP prevention strategies.

**Nomenclature**
AUC: area under the curve
CE: cardioembolic stroke
CI: confidence interval
GUSS: Gugging Swallowing Screen
LAA: large artery atherosclerosis
MST: nutritional risk screening tool
NCCT: noncontrast computed tomography
NGT: nasogastric tube
NIHSS: National Institutes of Health Stroke Scale
NLR: neutrophil‐to‐lymphocyte ratio
PR: proportion ratio
ROC: receiver operating characteristic
SAP: stroke‐associated pneumonia
SVO: small vessel occlusion
TOAST: Trial of Org 10172 in Acute Stroke Treatment
VESS: videoendoscopic swallowing study

## Author Contributions

L.A. conceived the study, performed data collection and analysis, drafted the manuscript, and submitted the manuscript. N.N.I. performed the data collection and analyzed the data.

## Funding

No funding was received for this manuscript.

## Disclosure

All authors read and approved the final manuscript.

## Consent

The authors have nothing to report.

## Conflicts of Interest

The authors declare no conflicts of interest.

## Supporting Information

Additional supporting information can be found online in the Supporting Information section.

## Supporting information


**Supporting Information 1** Bootstrap of internal validation analysis.

**Supporting Information 2** Figure_Bootstrap_Internal_Validation_SAP.png.

## Data Availability

The data that support the findings of this study are available from the corresponding author upon reasonable request.
