# Supplementary material for: Neutrophil‐to‐Lymphocyte Ratio as a Complementary Marker for Stroke‐Associated Pneumonia Risk in Acute Ischemic Stroke
Source: Stroke Res Treat. 2026 Sep 10;2026:1182945. doi: 10.1155/srat/1182945 (PMC13559792; doi:10.1155/srat/1182945)
Supplement: Supplementary file 1 [file SRAT-2026-1182945-s002.docx]

**Internal Validation and Bootstrap Analysis of the SAP Prediction Model**

The analysis used the supplied 615-patient dataset and reproduced the five predictors specified in the manuscript's final multivariable model: age >60 years, NIHSS >8, dysphagia, nutritional risk, and NLR >5.21.

# Methods – Internal validation

Internal validation was performed using non-parametric bootstrap resampling with 1,000 repetitions. For each bootstrap sample, the complete multivariable logistic regression model was refitted using the same five predictors. Model performance was calculated in the bootstrap sample and then evaluated in the original cohort. Optimism was defined as the difference between bootstrap-sample performance and performance in the original cohort, with the direction adjusted so that positive optimism represented apparent overperformance. Optimism-corrected estimates were obtained from the apparent performance after correction for the mean optimism. Discrimination was assessed using the area under the receiver operating characteristic curve (AUC/C-statistic), overall predictive accuracy using the Brier score, and calibration using the calibration intercept and slope. The calibration slope was additionally interpreted as a uniform shrinkage factor.

# Results – Internal validation

Among 615 patients, 121 (19.7%) developed SAP. The apparent five-predictor model showed an AUC of 0.918 and a Brier score of 0.086. After 1,000 bootstrap repetitions, mean optimism was 0.005 for the AUC, 0.003 for the Brier score, 0.021 for the calibration intercept, and 0.035 for the calibration slope. The optimism-corrected AUC was 0.913, while the optimism-corrected Brier score was 0.090. The optimism-corrected calibration intercept was -0.021, and the calibration slope was 0.965. The resulting uniform shrinkage factor of 0.965 indicates relatively limited overfitting within the derivation cohort.

# Table. Bootstrap internal validation of the multivariable SAP model

| Performance measure | Apparent | Mean optimism | Optimism-corrected | Bootstrap test-performance 95% CI |
| --- | --- | --- | --- | --- |
| AUC (C-statistic) | 0.918 | 0.005 | 0.913 | 0.911–0.919 |
| Brier score | 0.086 | 0.003 | 0.090 | 0.086–0.091 |
| Calibration intercept | 0.000 | 0.021 | -0.021 | -0.322–0.296 |
| Calibration slope | 1.000 | 0.035 | 0.965 | 0.800–1.144 |

Note: Lower Brier scores indicate better overall predictive accuracy. Calibration intercept values closer to 0 and calibration slopes closer to 1 indicate better calibration. The confidence intervals are the 2.5th and 97.5th percentiles of the bootstrap test-performance distribution.

# Discussion – Internal validation and model robustness

The bootstrap analysis provides evidence that the SAP prediction model retained good performance after correction for optimism. The apparent AUC of 0.918 decreased only modestly to an optimism-corrected AUC of 0.913, with mean optimism of 0.005. This relatively small optimism suggests that the discrimination observed in the derivation cohort was not substantially driven by overfitting. Internal validation is particularly important in a retrospective single-center dataset because the same observations are used for model estimation and initial assessment of performance.

Calibration was also preserved after bootstrap correction. The calibration intercept was -0.021, close to the ideal value of zero, while the calibration slope was 0.965, close to the ideal value of one. The corresponding shrinkage factor of 0.965 indicates only modest regression-to-the-mean correction of the model coefficients. Taken together, these findings suggest that the model combines adequate discrimination with acceptable calibration within the development population.

The model is clinically coherent because it combines different dimensions of SAP susceptibility: neurological severity (NIHSS), impaired airway protection (dysphagia), biological/inflammatory vulnerability (NLR), age-related risk, and nutritional risk. This integrated approach is consistent with the manuscript's proposed framework in which acute stroke-related systemic inflammation and immune dysregulation coexist with impaired airway protection and aspiration susceptibility. NLR therefore contributes complementary biological information rather than replacing clinical assessment of stroke severity or swallowing function.

However, the bootstrap results should not be interpreted as evidence of external validity. Internal validation evaluates model stability within the development cohort and cannot determine whether the same coefficients, risk thresholds, calibration, or discrimination will be maintained in other hospitals or populations. External validation using an independent cohort is therefore required before clinical implementation. Prospective validation should also use standardized timing of NLR measurement and standardized dysphagia assessment.

# Critical data–manuscript consistency check

An important discrepancy was identified when the supplied dataset was re-analysed using exactly the five predictors listed in the manuscript. The re-estimated ORs were 3.87 for age >60, 4.39 for NIHSS >8, 4.46 for dysphagia, 2.80 for nutritional risk, and 11.43 for NLR >5.21. These differ from the current manuscript Table 4 values (3.74, 4.28, 4.64, 2.76, and 10.40, respectively). Therefore, Table 4, the regression equation, and any prediction nomogram should be regenerated from the final locked dataset before submission.

The continuous-variable ROC results are consistent with the manuscript: AUC was 0.784 for continuous NLR, 0.835 for continuous NIHSS, and 0.666 for continuous age. Thus, the discrepancy concerns the multivariable regression coefficients rather than the individual continuous-variable ROC analyses.

# Suggested manuscript conclusion after internal validation

Internal bootstrap validation demonstrated that the multivariable model incorporating age >60 years, NIHSS >8, dysphagia, nutritional risk, and NLR >5.21 retained good discriminative performance after correction for optimism (optimism-corrected AUC 0.913). Calibration was acceptable, with an optimism-corrected calibration intercept of -0.021 and calibration slope of 0.965. These findings support the internal robustness of the model but do not replace external validation. NLR should therefore be interpreted as a complementary inflammatory biomarker within a multidimensional SAP risk model rather than as a standalone predictor.
